# Qualitative and Quantitative Comparison of Hippocampal Volumetric Software Applications: Do All Roads Lead to Rome?

**DOI:** 10.3390/biomedicines10020432

**Published:** 2022-02-12

**Authors:** Stephanie Mangesius, Lukas Haider, Lukas Lenhart, Ruth Steiger, Ferran Prados Carrasco, Christoph Scherfler, Elke R. Gizewski

**Affiliations:** 1 Department of Neuroradiology, Medical University of Innsbruck, Anichstrasse 35, 6020 Innsbruck, Austria; stephanie.mangesius@i-med.ac.at (S.M.); lukas.lenhart@i-med.ac.at (L.L.); ruth.steiger@i-med.ac.at (R.S.); elke.gizewski@i-med.ac.at (E.R.G.); 2 Neuroimaging Core Facility, Medical University of Innsbruck, Anichstrasse 35, 6020 Innsbruck, Austria; 3 NMR Research Unit, Queen Square Multiple Sclerosis Centre, University College London Institute of Neurology, Russell Square House, Russell Square 10-12, London WC1B 5EH, UK; f.carrasco@ucl.ac.uk; 4 Department of Biomedical Imaging and Image Guided Therapy, Medical University of Vienna, Währinger Gürtel 18-20, 1090 Vienna, Austria; 5 Centre for Medical Image Computing (CMIC), Department of Medical Physics and Biomedical Engineering, University College London, Malet Place Engineering Building, Gower Street, London WC1E 6BT, UK; 6 e-Health Centre, Universitat Oberta de Catalunya, Rambla del Poblenou 156, 08018 Barcelona, Spain; 7 Department of Neurology, Medical University of Innsbruck, Anichstrasse 35, 6020 Innsbruck, Austria; christoph.scherfler@imed.ac.at

**Keywords:** magnetic resonance imaging, brain, software, hippocampus, atrophy

## Abstract

Brain volumetric software is increasingly suggested for clinical routine. The present study quantifies the agreement across different software applications. Ten cases with and ten gender- and age-adjusted healthy controls without hippocampal atrophy (median age: 70; 25–75% range: 64–77 years and 74; 66–78 years) were retrospectively selected from a previously published cohort of Alzheimer’s dementia patients and normal ageing controls. Hippocampal volumes were computed based on 3 Tesla T1-MPRAGE-sequences with FreeSurfer (FS), Statistical-Parametric-Mapping (SPM; Neuromorphometrics and Hammers atlases), Geodesic-Information-Flows (GIF), Similarity-and-Truth-Estimation-for-Propagated-Segmentations (STEPS), and Quantib™. MTA (medial temporal lobe atrophy) scores were manually rated. Volumetric measures of each individual were compared against the mean of all applications with intraclass correlation coefficients (ICC) and Bland–Altman plots. Comparing against the mean of all methods, moderate to low agreement was present considering categorization of hippocampal volumes into quartiles. ICCs ranged noticeably between applications (left hippocampus (LH): from 0.42 (STEPS) to 0.88 (FS); right hippocampus (RH): from 0.36 (Quantib™) to 0.86 (FS). Mean differences between individual methods and the mean of all methods [mm^3^] were considerable (LH: FS −209, SPM-Neuromorphometrics −820; SPM-Hammers −1474; Quantib™ −680; GIF 891; STEPS 2218; RH: FS −232, SPM-Neuromorphometrics −745; SPM-Hammers −1547; Quantib™ −723; GIF 982; STEPS 2188). In this clinically relevant sample size with large spread in data ranging from normal aging to severe atrophy, hippocampal volumes derived by well-accepted applications were quantitatively different. Thus, interchangeable use is not recommended.

## 1. Introduction

Assessment of atrophy aids in distinguishing clinically and cognitively deteriorating subjects and allows prediction of those who will have a less favorable clinical outcome in various neurological diseases [1]. Hippocampal size can be measured from brain MRI scans with visual assessment [2,3], linear measurements [2,4], manual volumetry [4] and automated volumetry [3,5]. With the advance of precision medicine, numerous open source and commercial software applications have evolved to allow automated and thus potentially fast and unbiased measurement of brain volumes. To date, none of these approaches has emerged as a gold standard in clinical routine or research. Hence, the measurement of atrophy in routine clinical practice remains an unmet need. Additionally, while these applications have repeatedly been shown to be highly consistent within themselves when applied repeatedly to the same MRI acquisition, consistency has remained less clear when the same subject is scanned twice within the same imaging session using similar MRI parameters [6]. Even more, and this point is most relevant for consistency across both clinical care providers and across research groups, their relative performance against each other is rarely investigated. For reasons of availability of cerebral regions similarly segmented by all included applications, the analyses of the present study were limited to the hippocampus. While differences in other anatomical areas might have been smaller or larger, this is an anatomically well-defined and circumscribed area with overall good segmentation results. Further, the hippocampal volume is a biomarker for multiple neurological conditions [7], including major depressive disorder [8,9], epilepsy [7,10,11], post-traumatic stress disorder [12] and Alzheimer’s Disease [13,14,15], as well as normal aging [16,17,18,19,20,21], and is also one of the major brain sites of neuroplasticity [22]. We therefore aimed to quantify the extent of agreement between a set of well-established brain volumetric software applications (FreeSurfer (FS), statistical parametric mapping (SPM) using two different atlases, Quantib™, Geodesic Information Flows (GIF), and Similarity and Truth Estimation for Propagated Segmentations (STEPS)) in a sample size and an anatomical area that is relevant for a clinical setting.

## 2. Materials and Methods

The study was conducted in accordance with the Declaration of Helsinki and approved by the local Ethics Committee of the Medical University of Innsbruck (AN2016-0099). All participants provided written informed consent to participate in the study.

### 2.1. Study Population

FS has been additionally applied in our clinic for many years during diagnostic work up of patients with memory deficits, and measurements derived from this method were therefore chosen as inclusion criteria. Based on hippocampal z-scores < −1.96, measured by FS, we retrospectively selected 10 cases and 10 gender- and age-adjusted healthy controls without hippocampal atrophy from a previously published cohort of Alzheimer’s dementia patients and normal ageing controls [23,24]. Z-scores were derived by individually age- and gender-matched control datasets, which were characterized by normal cognitive functions determined by neuropsychological tests and had no history of neurological or psychiatric disorders with an age range of 44 to 85 years. Out of this healthy control cohort, sex-matched groups of at least 35 subjects with an age range of ±5 years of the individual subject to be analyzed was drawn to serve as healthy subjects’ sample to enable z-transformation of regional morphometric measures for every single study participant [25]. Z-transformations provide the fractional number of standard deviations, by which each observed value is above or below the mean value of a group. Additionally, 10 sex- and age-matched healthy controls (HC) were recruited prospectively. Subjects with evidence of structural brain lesions such as territorial ischemia, mass lesions, etc. were excluded.

### 2.2. Magnetic Resonance Imaging Protocol and Image Analysis

High-resolution isovoxel T1-weighted magnetization-prepared rapid gradient-echo (MPRAGE) sequences (TR = 2210 ms, TE = 3 ms, flip angle (FA) = 8°, field of view (FOV) = 220 mm× 179 mm, acquisition time (TA) = 3:37) were acquired for all individuals using a 3 Tesla MR-scanner (MAGNETOM Skyra, Siemens Healthcare GmbH, Erlangen, Germany) with a standard 64-channel head coil. MRI acquisition (scanner and parameters) for this dataset were consistent for all examined subjects.

### 2.3. Volumetric Measurements

Volumetric analyses were performed with the following five programs: FS, SPM applying two different atlases (Neuromorphometrics and Hammers), GIF, STEPS and the commercially available Quantib™. Volumetric analysis with FS was conducted using the software package version 6.0 (http://surfer.-nmr.mgh.harvard.edu (accessed on 12 December 2020), Harvard University, Boston, MA, USA). Data was further processed by z-transformation using mean centering and unit-variance scaling of in-house gender- and age- adjusted HC cohorts. Using SPM 12 (http://www.fil.ion.ucl.ac.uk/spm (accessed on 12 December 2020), Institute of Neurology, London, UK) the estimation of TIV was conducted while running MATLAB 9.5 (R2018b; MathWorks, Natick, MA, USA). For the extraction of hippocampal volumes, we used the manually annotated Neuromorphometrics atlases (Neuromorphometrics, Inc. under academic subscription, http://Neuromorphometrics.com (accessed on 12 December 2020)) and the Hammers atlas [26]. Quantib™ (Quantib B.V., Rotterdam, Netherlands) was used as instructed by the vendor and necessitated the import of data from our routine clinical image software via a locally already established data node only. GIF [27,28] and STEPS [29] required the export of anonymized image data and subsequent upload on a cloud-based server (http://niftyweb.cs.ucl.ac.uk/program.php?p=GIF (accessed on 12 December 2020), http://niftyweb.cs.ucl.ac.uk/program.php?p=BRAIN-STEPS (accessed on 12 December 2020). No pre- and postprocessing were necessary for the application of GIF and STEPS. Due to its clinical applicability, the visual MTA (medial temporal lobe atrophy) score was performed on MRI of the brain using coronal (reconstructed from isovoxel) T1 weighted images on a slice through the hippocampus at the level of the anterior pons for each hemisphere separately as reported previously [30,31]. The analysis was performed in consensus by S.M. and L.L. In case of disagreement, expert decision was considered (E.G.).

### 2.4. Statistical Analysis 

In a first step, subjects were assigned to quartiles (within all data available in this cohort) according to their volumetric measure for each method, in order to investigate, whether different software applications categorized them in the same quartiles. In a second step, volumetric measures of both hippocampi between each volumetric software application and the mean of all values were compared with intraclass correlation coefficients (ICC), implementing two-way consistency analysis. The comparison against the mean of all methods was chosen because of the lack of a generally accepted gold standard. In a third step, Bland–Altman statistics and plots were calculated to assess the amount of disagreement between methods across the spread of the data, again comparing against the mean of all methods.

## 3. Results

The median age in subjects selected based on low z-scores in our FS data base was 70 years (25–75% range: 64–77 years; f:m = 4:6) and 74 years in the control group (66–78 years; f:m = 5:5:). One subject could not be processed with Quantib™ due to software-related reasons but was otherwise assessed with all other applications. There was no visually perceivable image alteration such as image acquisition-related artefacts or structural brain lesions in this scan. Volumetric values in mm^3^ of all analyzed applications and the MTA scores are visualized in Table 1.

**Figure 1:**
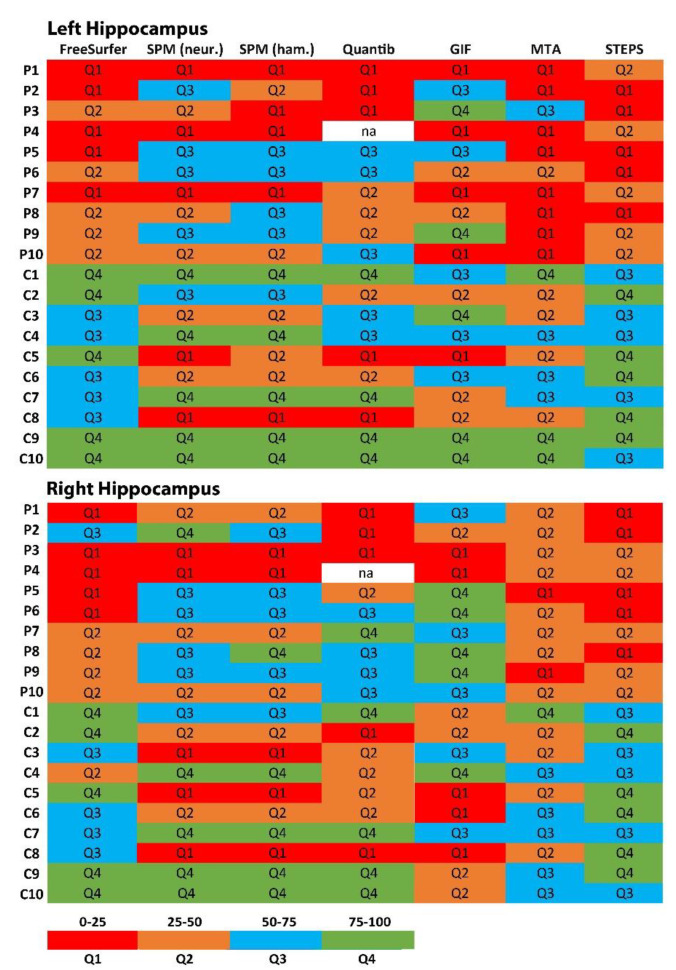
Attribution of left and right hippocampus to color-coded quartiles which were defined within each method. Legend: whether subjects are assigned to the same category by means of different software applications is visualized. For example, for subject P5 right hippocampus is assigned to Q1 in FreeSufer and STEPS, while the same structure is attributed to the highest quartile in GIF. Abbreviations: SPM = Statistical Parametric Mapping software; GIF = Geodesic Information Flows software; STEPS = Similarity and Truth Estimation for Propagated Segmentations.

Noteworthy, the observed differences between several methods were greater than the measurements themselves. The differentiation between the two groups (individuals selected via FS z-scores< −1.96 and matched HC) via quartile ratings was best reproduced by STEPS and MTA scores. SPM, Quantib™ and GIF have statistical outliers, as some HC are categorized in the quartile with the most atrophy. Quantib™ and GIF generally tend to categorize subjects to lower quartiles. Observations were nearly the same for both hemispheres (Figure 1).

All ICC were statistically significant with the exception of Quantib, which missed the preset level of statistical significance in the right hippocampus with 0.36 (95%CI: −0.10–−0.69), *p* = 0.059. The highest ICC was reached by FS in the left hippocampus with 0.88 (95%CI: 0.73–0.95), *p* < 0.001 and the right hippocampus with 0.86 (95%CI: 0.68–0.94), *p* < 0.001. The second highest ICC was reached by SPM (Neuromorphometrics) in the left hippocampus with 0.73 (95%CI: 0.44–0.89), *p* < 0.001 and the right hippocampus with 0.62 (95%CI: 0.25–0.83), *p* = 0.001 (Table 2).

**Figure 2:**
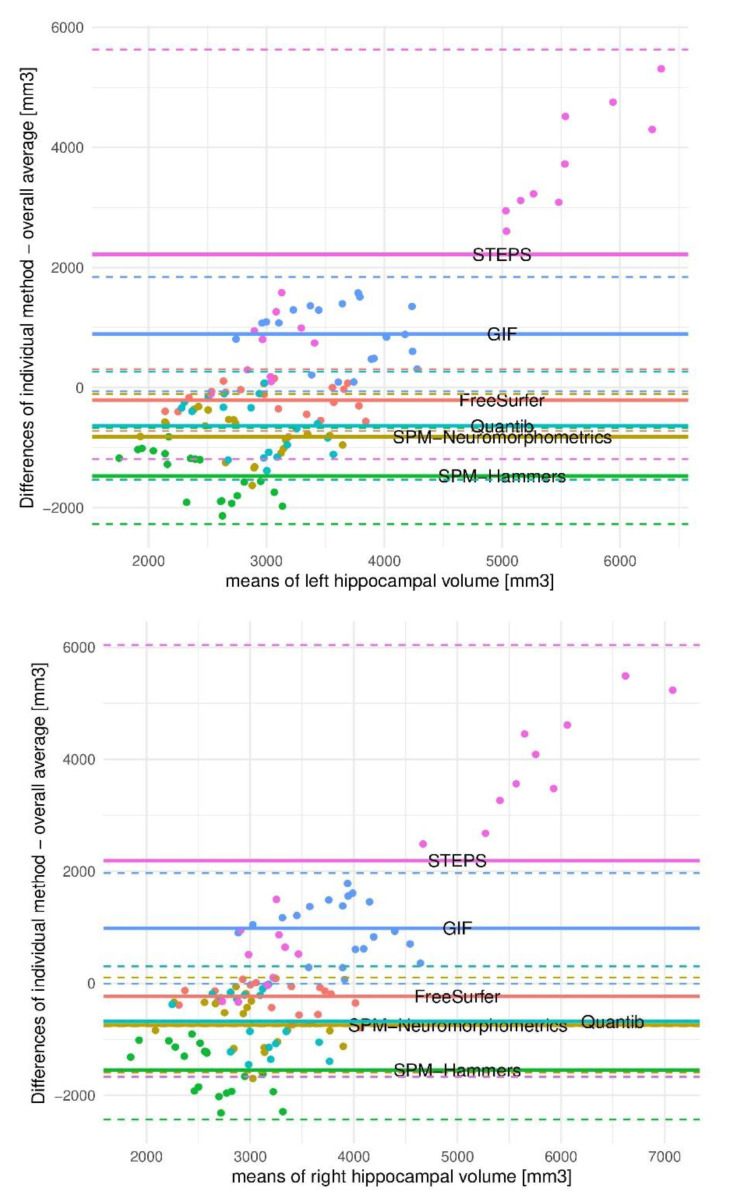
Bland–Altman plots of the relation of hippocampal volumetric measurements resulting from one single method to the overall methods. Legend: The transversal color-coded continuous line parallel to the x- axis visualizes the mean of differences of single method means to the overall mean. A line along the 0 values would be the optimum, as it is near the mean of all methods. The discontinuous line depicts the limitations of agreement, which varies substantially between the methods. STEPS reveals a great spread in data and measures the highest values compared with the mean. However, this method forms two clusters, one including subjects, the other controls, therefore yielding a good separation between pathological and normal. Abbreviations: SPM = Statistical Parametric Mapping software; GIF = Geodesic Information Flows software; STEPS = Similarity and Truth Estimation for Propagated Segmentations.

In the Bland–Altman plots (Figure 2) the means of left and right hippocampal volumes were plotted against the differences of the individual method minus the overall mean of all methods, to visualize the relation of one single method to the overall methods. Measures from Quantib™ and SPM Neuromorphometrics were closely similar. Both SPM measures using Neuromorphometrics and Hammers were below the group mean. Volumetric estimates from FS were closest to the mean measure. Values obtained from GIF and STEPS were above the mean, with highest values measured in the latter. Mean differences between individual methods and the mean of all methods in mm^3^ was considerable (LH: FS −209, SPM-Neuromorphometrics −820; SPM-Hammers −1474; Quantib™ −680; GIF 891; STEPS 2218; RH: FS −232, SPM-Neuromorphometrics −745; SPM-Hammers −1547; Quantib™ −723; GIF 982; STEPS 2188).

## 4. Discussion

Brain atrophy occurs in various neurological diseases and is one of the best investigated imaging biomarkers, due to its promising correlation with present and future disability [1]. Important technical improvements for quantification of brain atrophy have been achieved and several software applications, with differing requirements on technical ability and levels of operator intervention, have been developed. Despite extensive research, their application in clinical routine settings is limited.

This is in part due to small group differences that become apparent on a group basis but provide limited applicability on a patient level [32,33]. To some extent, it also reflects the fact that comparative studies between different methods are sparse [34]. It is thus unknown to what extent different software applications agree regarding the same anatomical areas [35]. This issue is not only of academic interest, as volume segmentation in different software products may lead to significantly different results in the individual patient and may thus seriously influence therapeutic decisions, as was recently shown for automated MRI perfusion-diffusion mismatch volume estimation and the consecutive decision for or against mechanical thrombectomy [36]. In this study, we therefore investigated the quantitative agreement between well-established volumetric applications in a well-separated cohort and found major differences.

There are several freely available and commonly applied tools for brain volumetry including FS, SPM, Quantib™, GIF and STEPS. These software programs can automatically pre-process and segment T1-weighted images of the brain. FS combines volumetric- and surface-based approaches and uses a computationally demanding, template-driven approach to provide a detailed parcellation and segmentation of cortical and subcortical structures [37]. SPM is computationally less demanding and based on spatial normalization of the individual brain in the same stereotactic space (Montreal Neurological Institute (MNI) space), which allows the segmentation of brain tissues by assigning tissue probabilities per voxel [38]. For voxel-based ROI extraction, SPM offers a selection of volume-based atlases in the predefined template space [39]. Quantib™ is a commercially available software, which implements a fully automated brain tissue classification procedure, in which k-Nearest-Neighbor (kNN) training is automated. This is achieved by non-rigidly registering the MR data with a tissue probability atlas to automatically select training samples, followed by a post-processing step to keep the most reliable samples [40,41,42]. GIF algorithm is a brain extraction, tissue segmentation and parcellation tool, which assumes probabilities for a specific voxel to belong to a certain brain structure [27,28]. STEPS is a multi-atlas segmentation propagation and fusion technique that generates probabilistic masks using a template library with associated manual segmentations [27,29].

Both, FS and SPM, are scientifically well-established software programs. FS has been additionally applied in our clinic for many years during diagnostic work up of patients with memory deficits. FS and SPM have been extensively used at our center in various studies, and therefore a profound knowledge of these programs is present in our team [23,24,43,44,45,46]). Quantib™ was chosen as an example of a commercially available software program and was provided to us during a trial period. GIF [27,28] and STEPS [29] were chosen as they are server-based non-commercial tools for which no preprocessing is necessary, and the raw exported and anonymized data are processed on a cloud-based server. The research of MR volumetric imaging markers for neurodegenerative disease, especially of those resulting in cognitive decline, [47], and their potential bias induced by the choice of method [48,49] are of ongoing major interest in both, clinical and scientific communities. Advances in neuroimaging techniques have contributed greatly to the development of novel morphometric methods [50]. Automated imaging techniques, such as SPM, have led to the possibility of characterizing neuroanatomical structures and measuring regional brain alterations in aging, learning, development and neurodegenerative diseases [51]. Quantitative MRI analysis was shown to be useful for the radiological assessment of altered brain structures when implemented in the clinical routine workflow [52]. As regional cerebral atrophy is typically associated with neurodegenerative diseases, quantitative brain measures such as SPM have been utilized as an independent morphometric biomarker to evaluate morphometric changes in the structure of the premorbid brain [53,54,55,56,57]. SPM has been used for the discrimination of Alzheimer’s disease from cognitively normal population [49] and for the detection of atrophy patterns in the premorbid brain of Alzheimer’s disease patients [58]. Along with age and gender, TIV is an important covariate that should be corrected for in regression analysis investigating progressive neurodegenerative brain disorders, such as Alzheimer’s disease, normal aging and cognitive impairments [59]. While a very prominent and scientifically applied function of FS is whole-brain segmentation [60,61], FS is constantly being extended with updated tools for accurate cross-modal intra-subject registration [62], combined volume and surface cross-subject registration [63], probabilistic estimation of cytoarchitectonic boundaries [64], automated tractography [65], and longitudinal analysis [66,67]. It has further enabled the comprehension of many neurological disorders [37], the genetic influence of neuroanatomical diversity and change [68,69], physiological development [70] as well as the underlying process of aging [71]. The Quantib™ algorithm has been evaluated and applied in studies focusing on cognitive impairment and dementia, and further cerebral small vessel disease [72,73,74]. GIF [27,28] and STEPS [29] use a template library with associated manual segmentations including 682 brain and 110 hippocampal manual segmentations, which makes it reliable for hippocampal segmentations and could thus also be considered as an alternative to manual segmentations by the user.

In this study, image acquisition, processing and volumetric applications were performed according to current scientific standards. While all volumetric applications under consideration in the present study are scientifically well established and highly consistent within themselves, there is no generally accepted automated MR volumetric gold standard [33]. We therefore operationalized the mean of all values to be closest to the unknown ground truth.

In a first step, we asked a clinically relevant question, namely, to which extent different applications attribute subjects concordantly into the same categories of atrophy. Patients and controls were best separated in this approach by FS and STEPS. In a second step, we investigated whether all methods correlate with each other, and found that highest correlations with the mean of all groups was present for FS and SPMS. In the last step, the extent of absolute volumetric differences was quantified with Bland–Altman statistics. We found that the differences between some absolute values were larger than the measurement themselves e.g., in the healthy control (C2), STEPS revealed a hippocampal volume of 7395 mm^3^ and FS of 3643 mm^3^. Generally speaking, results obtained by Quantib™ and SPM are close to each other, FS is close to the overall mean with the smallest deviation from zero value, STEPS “overestimates” the value, SPM Hammers “underestimates” the value. However, the zero line, reflecting the mean of all values, might change depending on the potential for an additionally applied method and atlas.

Likely, this reflects the underlying segmentation protocols that include different anatomical areas under the term “hippocampus”. The Dementia Research Centre protocol used for STEPS includes the dentate gyrus, the hippocampus proper, the subiculum and the alveus. Contrarily, the protocol used for GIF cuts the tail of the hippocampus when the tail turns dorsally (“Crura and Tail End”) [27]. While the investigation of such differences is not the subject of the current investigation, it does point to the fact that serious differences are present in areas that are considered clearly defined from a neuroradiological point of view.

In our present study, we observed larger hippocampal volumes measured by FS and STEPS, compared with SPM or Quantib™. This is in line with a large multicenter observational study, which reported that absolute ROI volumes of total intracranial volume, total white matter and grey matter volume, total ventricular volume, right and left volumes for the basal ganglia, amygdala and hippocampus derived from FS 6.0 differed significantly from those obtained using version 5.3 [75]. FS consistently reports larger volumes than manual tracing. This difference is smaller in larger hippocampi or older people, with weaker biases in version 6.0.0 than prior versions. All methods tested agree qualitatively on rightward asymmetry and increasing atrophy in older people. FS approximates the same atrophy measures as manual tracing, but it introduces biases that could require statistical adjustments in some studies.

While reliability between the two segmenting tools NeuroQuant^®^ and FS is fair to excellent, volumetric outcomes are statistically different between the two methods [76]. Due to these known observations, as suggested by developers of FS and NeuroQuant^®^, structure segmentation should be visually verified prior to clinical use and rigor should be used when interpreting results generated by either method [76]. We have recently shown that MR planimetric measurements are highly predictive for volumetric measurements, thus even if absolute measurements of cerebral atrophy are different between volumetric software applications, this finding does not mean that one method could not predict another.

A clinically feasible method for the evaluation of medial temporal lobe atrophy that is useful in diagnostic work-up of Alzheimer’s disease is the medial temporal lobe atrophy (MTA) score, which was shown to be equally good regarding diagnostic properties to volumetric measurements [77]. In subjects with Alzheimer’s dementia, and clinically non proven forms of dementia (non-dementia), the NeuroQuant^®^ total measure yielded a comparably higher AUC (0.88, “good”) compared with the MTA mean measure (0.80, “good”) in the comparison of subjects with Alzheimer’s disease and non-dementia. The accuracy, however, was in favor of the MTA scale. Therefore, both methods reached equally “good” power and correlated highly with each other [77]. Contrarily to Quantib™, MTA categorized the subjects in quartiles similarly to FS and STEPS.

This study has several limitations. First, there is no gold standard to compare with. While the comparison against the mean of all groups is likely to include a fairly appropriate estimate of the ground truth based on the inclusion of five well-established applications, the inclusion or exclusion of applications clearly exerts a strong bias. However, as inclusion or exclusion of other applications will shift the mean and change the correlation coefficients or render their significance levels, it does not affect the observation that there are major differences in the absolute values between these different key applications, and we do not draw any conclusions form our data that exceed this fact. We do point out in this context that the software applications considered in this manuscript, while representative, are not entirely exhaustive as several, especially commercially available, applications were not included.

Second, sample size is small in absolute numbers, but highly representative for a memory clinic setting, where decisions are made on an individual subject basis and not on large sample sizes. As the discussion is currently moving towards integrating MR volumetric tools in the clinical setting, the observed differences in this cohort cannot be neglected irrespective of the sample size. Contrarily, it is likely that our cohort of 10 subjects with severe hippocampal atrophy and 10 healthy controls will oversimplify any diagnostic test to separate the two groups. As this separation was largely absent in our derived data set, it is likely that in a cohort with less pronounced group differences, the agreement would be even weaker than reported here, especially considering the fact that confounding factors such as structural brain lesions were excluded in the present analysis. Furthermore, while correlations across methods would increase with sample size, we consider it highly relevant to point out that on an individual patient level this association is obviously not given, and methods should not be used interchangeably.

Patients typically receive scans at different institutions, and with the advance of volumetric tools in clinical practice it is likely that a patient will be confronted with reports providing significantly different values for the same MR scan. We believe that it is important for the research community to be aware of this, and to transport this message to clinicians.

While FS leads in our investigation concerning concordance with the overall means, we cannot conclude whether this is due to superior performance or simply due to the fact that subjects were initially recruited based on z-scores obtained from FS segmentations. Potentially, measurement errors from FS-derived volumes have contributed to false misclassification of this cohort as having low hippocampal volume. FS was chosen as an instrument for applying inclusion criteria, as this software program has been additionally applied in our clinic for many years during diagnostic work-up of dementia.

It is, however, important to stress at this point, that this study does not intend to support one method or the other, but merely to point out a major issue regarding variability in volumetry. One case could not be analyzed with Quantib^TM^, which further limited the sample size for the comparison including this method. We, however, did not exclude this case from the analysis, as there were no visually perceivable reasons for this, such as image acquisition-related artefacts or structural brain lesions.

In this study, we used a large, but finite, number of volumetric methods and certain methods, including manual segmentations, were not included. The DRC hippocampus volumetry is, however, based on expert hippocampal segmentations, and FS approximates the same atrophy measures as manual tracing [78].

ICC were calculated based on the mean of a single method and the mean of all methods. This calculation results in the mean of the method being represented in the mean of all methods, thereby increasing the consistency of the two measurements and potentially overestimating the amount of agreement. Another possibility would have been comparing the mean of a single method to the mean of the other five methods included. The reason for choosing the reported approach of method comparison is that, by including all methods at all times, we gain a homogeneous “mean method/surrogate gold standard” across all comparisons throughout the entire analysis. The alternative approach would create six different “surrogate gold standards“ by always omitting the method compared, consequently hindering comprehensive presentation and interpretation. Furthermore, given the presumption that the methods investigated cover the ground truth, the true mean should contain the method under investigation. Otherwise, if we would not suppose that a certain method could potentially cover the ground truth, it should not be included in the analysis anyhow, especially not for “surrogate gold standard“ calculations serving as comparison for other methods.

As the specific research question of this manuscript is to quantify the amount of agreement across well-established software applications in their assessment of hippocampal volume within the same data set, we did not focus on other related aspects such as usability, hardware requirements, reproducibility with varying acquisition parameters, patient hydration status and cardiac output, the presence of structural brain alterations, or different imaging time points [79]. However, all those factors will play a considerable role in the real-life application of volumetric brain analysis and are currently poorly controlled for. It is thus likely that our study significantly overestimates the amount of agreement between volumetric software applications that will be encountered in a clinical setting.

The compared software packages apply different segmentation algorithms for calculation of the hippocampal volume. The exact underlying algorithm which might potentially influence measurements is often not known [36]. Since the application of such software programs in clinical routine is regarded to be without user interaction, the missing in-depth comprehension of the underlying algorithms does not influence the results of our study. Lastly, we did not attempt to comment on clinical applicability. In general, non-commercial software programs tend to require more expenditure of work and more experience and training compared with commercial software solutions. The time to produce individual reports, however, will depend on computer skills and computational resources. Hence, computation times might vary depending on the infrastructure.

The aim of our study was to measure the amount of agreement, yet we found significant disagreement. Any radiologist who would want/need to compare measurements across volumetric methods, such as during follow-up examinations, should be aware of this, and maybe consider using a mix of them. In the end, it is, however, irrelevant if the mean of all methods (which of course is arbitrary based on the included methods) does or does not outperform individual methods.

If one specific method would indeed outperform the mean of all methods, yet still not establish the ground truth, we could still not reliably conclude that the use of a mix of well-established methods is inferior to this single method. Especially as we now know that the real issue lies in inter-software disagreement, and therefore refrain from commenting on the accuracy of one or the other. Further, assuming a physiological loss of brain volume of about 0.3% per year in healthy adult subjects [80], which may even double in some neurological diseases [81,82], even with a volumetry software program with the highest accuracy, reliable estimation of brain atrophy in individual patients has been suggested to only be possible over periods of at least five years [83]. Considering the substantial disagreement between software programs for longitudinal patient follow-up, the expected effect size of hippocampal atrophy should exceed the size of differences between individual methods observed in this study.

## 5. Conclusions

Consistency across centers is viable for any diagnostic test. In the view of our finding and the lack of a generally accepted gold standard in the foreseeable future, we suggest the implementation of a spectrum of measurements obtained from a set of applications, rather than of focusing on a single solution.

## Figures and Tables

**Table 1:** Demographic and volumetric data of subjects with hippocampus volume loss and healthy controls.

ID	Age [y]	Gender	Free Surfer z-Value		FreeSurfer [mm^3^]		SPM Neuromorphometrics [mm^3^]		SPM Hammers [mm^3^]		Quantib™ [mm^3^]		GIF [mm^3^]		STEPS [mm^3^]		MTA	
			LH	RH	LH	RH	LH	RH	LH	RH	LH	RH	LH	RH	LH	RH	LH	RH
P1	68	m	−3.45	−2.42	2258	2593	1852	2390	1393	1703	2180	2540	3499	3896	3368	3242	3	2
P2	65	f	−3.03	−1.33	2615	3063	2318	2868	1588	1982	2590	2840	4053	4831	2985	2718	3	2
P3	74	f	−1.82	−2.42	2500	2307	2204	2097	1512	1421	2170	2060	3642	3335	3368	3383	1	2
P4	71	f	−4.59	−4.34	1942	2119	1522	1667	1161	1190	-	-	3146	3548	3920	4001	3	2
P5	58	m	−3.33	−2.64	3009	3279	2414	2756	1765	1964	3020	3170	4341	4793	3124	3149	3	3
P6	61	m	−2.66	−2.79	3142	3136	2454	2855	1799	1953	2890	3070	4567	4723	3088	3274	2	2
P7	81	f	−3.12	−2.17	2048	2527	1848	2485	1437	1709	2110	2590	3545	4053	3713	3711	3	2
P8	66	m	−2.79	−2.27	2688	2966	2265	2833	1761	1987	2430	2730	3874	4259	2471	2571	3	2
P9	77	m	−2.14	−1.65	2922	3293	2440	2664	1834	1961	2700	2990	4547	4583	3779	3728	3	3
P10	77	m	−2.27	−1.92	2764	2989	2159	2492	1520	1714	2470	2730	4089	4502	3791	3661	3	2
C1	81	f	1.79	0.81	3725	3653	2636	2742	1851	1857	2700	2710	4126	4404	6880	7348	0	0
C2	74	m	0.71	0.67	3643	3636	2576	2563	1740	1688	2510	2260	4155	4319	7395	7798	2	2
C3	74	m	−0.19	−0.48	3559	3371	2240	2244	1662	1578	2480	2570	4910	4879	6504	5911	2	2
C4	71	m	−1.23	−1.41	3186	3376	2961	3215	2169	2312	3130	3220	4618	4859	6338	6610	1	1
C5	82	m	1.13	1.15	3447	3685	2063	2178	1558	1561	2310	2520	3787	3947	9005	9366	2	2
C6	76	f	−0.47	−0.22	3118	3189	2225	2524	1677	1793	2390	2610	3654	4034	8318	8366	1	1
C7	77	m	−0.38	0.23	2776	3039	2728	2942	2023	2117	2910	2920	4439	4606	6715	7042	1	1
C8	74	f	0.08	0.35	2923	2991	2027	2259	1365	1501	2070	2200	3488	3708	7796	7875	2	2
C9	49	f	0.98	1.23	3561	3671	3169	3336	2147	2171	3010	3070	4434	4824	8423	9695	0	1
C10	49	f	0.44	0.70	3631	3840	3137	3346	2194	2256	3100	3140	4540	4895	7024	7667	0	1

Legend: P(1–10) subjects with hippocampal z-scores < 1.96 in our FS database (highlighted in grey); C(1–10) = matched healthy controls. Abbreviations: m = male; f = female; LH = left hippocampus; RH = right hippocampus; SPM = Statistical Parametric Mapping software; GIF = Geodesic Information Flows software; STEPS = Similarity and Truth Estimation for Propagated Segmentations; MTA = medial temporal lobe atrophy score.

**Table 2:** Intraclass correlation coefficient between the mean of a single method and the mean of all methods.

	Method	ICC	Lower CI	Upper CI	*p*-Value
LH	FreeSurfer	0.88	0.73	0.95	<0.001
	SPM Neuromorphometrics	0.73	0.44	0.89	<0.001
	SPM Hammers	0.58	0.20	0.81	0.003
	Quantib™	0.49	0.05	0.76	0.015
	GIF	0.57	0.18	0.80	0.004
	STEPS	0.42	−0.02	0.72	0.030
RH	FreeSurfer	0.86	0.68	0.94	<0.001
	SPM Neuromorphometrics	0.62	0.25	0.83	0.001
	SPM Hammers	0.48	0.06	0.76	0.013
	Quantib™	0.36	−0.10	0.69	0.059
	GIF	0.54	0.13	0.79	0.006
	STEPS	0.38	−0.07	0.70	0.046

Abbreviations: LH = left hippocampus; RH = right hippocampus; SPM = Statistical Parametric Mapping software; GIF = Geodesic Information Flows software; STEPS = Similarity and Truth Estimation for Propagated Segmentations; ICC = intraclass correlation coefficient; CI =confidence interval.

## Data Availability

The authors take full responsibility for the data, the analyses and interpretation, and the conduct of the research and have full access to all of the data, of which we have the right to publish any and all data in the absence of a sponsor. Anonymized data, not published in the article, will be shared on reasonable request from a qualified investigator upon agreement with the local ethics committee.
